# 3D point cloud lossy compression using quadric surfaces

**DOI:** 10.7717/peerj-cs.675

**Published:** 2021-10-06

**Authors:** Ulfat Imdad, Mirza Tahir Ahmed, Muhammad Asif, Hanan Aljuaid

**Affiliations:** 1 Department of Computer Science, National Textile University, Faisalabad, Pakistan; 2 Department of Electrical and Computer Engineering, Queen’s University, Kingston, Canada; 3 Computer Sciences Department, College of Computer and Information Sciences, Princess Nourah bint Abdulrahman University (PNU), Riyadh, Sudia Arabia

**Keywords:** Virtual interest point, Registration, Point cloud

## Abstract

The presence of 3D sensors in hand-held or head-mounted smart devices has motivated many researchers around the globe to devise algorithms to manage 3D point cloud data efficiently and economically. This paper presents a novel lossy compression technique to compress and decompress 3D point cloud data that will save storage space on smart devices as well as minimize the use of bandwidth when transferred over the network. The idea presented in this research exploits geometric information of the scene by using quadric surface representation of the point cloud. A region of a point cloud can be represented by the coefficients of quadric surface when the boundary conditions are known. Thus, a set of quadric surface coefficients and their associated boundary conditions are stored as a compressed point cloud and used to decompress. An added advantage of proposed technique is its flexibility to decompress the cloud as a dense or a course cloud. We compared our technique with state-of-the-art 3D lossless and lossy compression techniques on a number of standard publicly available datasets with varying the structure complexities.

## Introduction

A rapid growth in the 3D sensing industry has enabled possibilities of 3D sensors in smart hand-held mobile as well as head-mounted devices. Similarly, autonomous vehicle industry is also using significant amount of depth sensors to capture metric information on the roads. 3D point cloud data requires more space as compared to image data due to additional dimensions which have the advantage of additional information. Therefore, 3D captured scenes are also useful in many applications, such as robotics, medicine, entertainment industry and provide the basis for rapid modeling in architecture, agriculture, construction of tunnels, industry automation and urban & regional planning. However, it is a challenging task to manage such a huge amount of data given the limited bandwidth and storage space for real-time point cloud transferring applications and to store it for further processing, respectively. Therefore, the increasing use of such devices requires techniques to manage 3D point cloud data efficiently and economically to not only save disk space but also to reduce the bandwidth requirements to transfer data. Various 3D compression techniques are studied in the literature, which can be classified into two major categories such as *lossy compression* and *lossless compression*. The lossless compression techniques can decompress point cloud into its original data points as without any loss of information (Mongus & Žalik, 2011; Moreno, Chen & Li, 2017; Garcia & de Queiroz, 2017). Such techniques are used to compress and decompress point cloud where the original point cloud is required after reconstruction and a minor alteration is not acceptable (Thanou, Chou & Frossard, 2016; Dickie, 2018). On the other hand, the lossy compression techniques reconstruct the point cloud aiming to maintain the structure and geometry on the compromise of minute details present in the original point cloud (Navarrete et al., 2016; Navarrete, Viejo & Cazorla, 2018; Trejos et al., 2018). The compression rate in lossy techniques is significantly higher as compare to lossless techniques because such techniques remove considerable information from the original data. The reconstructed point cloud is approximately similar to the original point cloud but not exact (Bletterer et al., 2016; Klima et al., 2016; Schmaltz et al., 2014; Morell et al., 2014; Zhang, Florêncio & Loop, 2014). Thus lossy compression techniques are used in applications that can tolerate the difference between decompressed and original point cloud data such as the feature extraction techniques depending on the geometry of the objects visible in a view (Ahmed, Marshall & Greenspan, 2017; Ahn et al., 2015; Thanou, Chou & Frossard, 2016; de Queiroz & Chou, 2016). In this work, we propose a novel lossy compression technique that employs the geometric information of the scene using quadric surface representation. We used quadric surface representation to extract the geometry of the point cloud. Our work is inspired by Birdal et al. (2018) and Ahmed, Marshall & Greenspan (2017). The point clouds under consideration are normally acquired from a commercial off-the-shelf 3D range sensor. Thus, the representation is 2.5D. A type of quadric surfaces, known as bivariate quadric surface, are capable enough to capture such representation with a minimal set of coefficients (Kukelova, Heller & Fitzgibbon, 2016). Thus, the quadric surface represents the underlying geometry of the point cloud. The point cloud is divided into several small segments using point normal and curvature clues. Then we compute the quadric surface coefficients of each segment as discussed in Section “RANSAC QFit Parameters” along with the boundary conditions. Thus the compressed information is based on only the quadric coefficients as well as the boundary condition.

The mathematical foundation of the algorithm makes it capable to decompress the point cloud with an adjustable point density at an acceptable root-mean-square error (RMSE) and processing time. The proposed technique is compared with several techniques on multiple datasets. However, the most relevant state-of-the-art two lossless (Burrows & Wheeler, 1994; Ziv & Lempel, 1977) and four lossy techniques (Navarrete, Viejo & Cazorla, 2018; Navarrete et al., 2016; Morell et al., 2014; Kammerl et al., 2012) techniques results are discussed and presented in the paper. The comparison is made on the basis of compression rate, root-mean-square error (RMSE), and processing time. Moreover, the proposed technique is compared using a well-known, published, and publicly available point cloud dataset, presented as a benchmark by several authors (Morell et al., 2014; Navarrete et al., 2016; Navarrete, Viejo & Cazorla, 2018). The dataset comprising three different categories of the complexity of the structure such as high, medium, and low.

The rest of the paper is organized as section Related Work shows a brief overview on a number of compression techniques. The compression and decompression methodology is presented in Methodology section. A detailed empirical comparison of the proposed technique with other related techniques is given in Experimental Results and Conclusion section concludes the whole work with limitations and future directions.

## Related Work

Due to readily available off-the-shelf 3D range sensors and their use in hand-held devices, head-mounted displays for various purposes such as augmented reality, face recognition, etc. Real-time processing, as well as transmission of 3D data, is a need. Researchers from all across the globe have developed several techniques on lossless as well as lossy compression. A brief overview of the state-of-the-art compression technique is presented in this section.

Schnabel & Klein (2006) presents the octree-based point cloud compression technique for lossy compression based on the concept of a double buffering octree data structure to detect and exploit temporal and spatial changes in point cloud data. The concept is based on representing the redundant patches from the point cloud with minimal information. The author claims to achieve real-time compression and decompression of the point cloud with a reasonable compression rate at a low reconstruction error.

Edge Enhancing an isotropic Diffusion (EED) is an outstanding technique to compress 2D images. This mechanism is further extended and applied to point cloud by Schmaltz et al. (2014). Instead of projecting the 3D data to a 3D plane and compressed it using the image compression technique. The point cloud data is sliced and redundant information of the headers is reduced. However, the compression ratio, in this case, is directly proportional to the Mean Square Error (MSE).

A geometry-based point cloud compression technique is propounded by Morell et al. (2014). They use the Delaunay triangles efficiently to preserve scenes. This method provides a fast and realistic scene reconstruction. However, the principle is to detect plane surfaces and then decompose the planer surfaces by using Delaunay triangles. The triangles are then used to compress and decompress. In the decompression phase, a uniform set of points is generated inside each triangle. Finally, each set of points is merged to reconstruct the whole scene. This method performs outstanding and achieves a high compression rate where the point cloud is composed of planer surfaces. However, the performance suffers when the point cloud is composed of non-planer surfaces. For such datasets, the non-planer surfaces are first decomposed into a large set of planer surfaces and then compressed using the triangulation framework. Due to a large set of triangles, the compression rate is very low.

Navarrete et al. (2016) presented a dataset for testing 3D data compression methods, known as 2DCOMET. The data set contains different structure and texture variability to evaluate the results. They also presented a compression technique to compress and register a point cloud using GMMs (Navarrete, Viejo & Cazorla, 2018). Points are selected and grouped, using a 3D-model based on planar surfaces. A fast variant of Gaussian Mixture Models and an Expectation-Maximization algorithm to replace the points grouped in the previous step with a set of Gaussian distributions. The models are then used for compression and decompression. Results are evaluated based on the compression ratio and root-mean-square error between the original and reconstructed point cloud. They claimed that 85.25% Compression ratio is achieved with 0.01 RMSE and the results are compared with other lossless and lossy techniques as well.

The methods presented by Morell et al. (2014) and Navarrete, Viejo & Cazorla (2018) have a major limitation of using planer representation as a starting point. In our work, we used quadric surfaces instead of planer surfaces to efficiently and effectively model non-planer and abstract surfaces captured by 2.5D sensors with bivariate quadric and full 3D surfaces with general quadric. The difference between the two representations of quadric is explained in the next section. The process in our technique is simplified by segmenting the point cloud considering the geometric information using point normal and curvature, and then quadric surface coefficients and boundary conditions of each segment are extracted. The coefficients and boundary conditions are used to compress and decompress the data. We tested our algorithm on the standard dataset as given by Navarrete et al. (2016) and results are compared based on three qualitative measures *i.e*., compression ratio, root-mean-square error, and processing time. The advantage of the proposed method is that it cannot only reconstruct point cloud very efficiently similar to the original point cloud with minimal error but also with an adjustable degree of data density *i.e*., resolution.

## Quadric Surface Representation

Ahmed et al. (2021) clarified that a quadratic surface is portrayed by a verifiable condition of degree two in ${\open R}^{3}$. It can be isolated into two bunches: *general* and *bivariate* quadric. General group speaks to volumes with three factors each of degree one and bivariate quadric represents surfaces and utilized to speak to actually demonstrate 2.5D point clouds with condition of three factors, two of which are of degree two and the other one variable of degree one. In 2.5D clouds, points are projected along a specific dimension (generally Z) and no points are projected on top of other points. This generally means that the shape has only one side (*e.g*. a mountain seen from the top, etc.).

A bivariate quadric is spoken to certainly as:

(1) $$p^TQp \left \{\matrix{ \gt  0  \iff  p \ \text{lies\ Above\ the\ surface} \cr = 0  \iff  p \ \text{lies\ on\ the\ surface} \cr \lt 0  \iff  p \ \text{lies\ below\ the\ surface}}\right.$$

where *p* may be a homogeneous 3D point and *Q* could be a 4 × 4 matrix called the *discriminant* of the quadric surface. Fulfilment of Eq. (1) certainly decides membership of point *p* on the quadric surface characterized by *Q*. Extending the components of Eq. (1) gives:

(2) $${\left[ \matrix{ x  y  z  1} \right] } { \left [ \matrix{  a  b  c  d \cr b  e  f  g \cr c  f  h  k \cr d  g  k  j } \right]} \left[ {\matrix { x \\ y \\ z \\ 1}} \right]} = 0} .$$

The upper-left 3 × 3 foremost submatrix of *Q*, termed the *subdiscriminant Q*_*u*_, contains all the second-order terms:

(3) }{}Q_u = \left[ {\matrix {a \quad b \quad c \\ b \quad e \quad f \\ c \quad f \quad h}} \right]

The positions of *Q* and *Q*_*u*_, in conjunction with the sign of the determinant of the discriminant det(*Q*), are supportive in classifying the quadric surface. There are 17 standard sorts as recorded in Table 1, with the planar, elliptic paraboloid and hyperbolic paraboloid types being well-suited to the 2.5D point cloud representation.

**Table 1:** Quadric surfaces type with their canonical equation is given for rank of discriminant Δ = *rank*(*Q*) and subdiscriminant Δ*_u_* = *rank*(*Q_u_*), and sign of the determinant of discriminant ρ = *sgn*(det(*Q*)).

Surface type	Equation	Δ	Δ_*u*_	*ρ*
Coincident Plane	*x*^2^ = 0	1	1	
Parallel Planes (imaginary)	*x*^2^ = − *a*^2^	2	1	
Parallel Planes (real)	*x*^2^ = *a*^2^	2	1	
Intersecting Planes (imaginary)	$\frac{x^2}{a^2} +\frac{y^2}{b^2} = 0$	2	2	
Intersecting Planes (real)	$\frac{x^2}{a^2} -\frac{y^2}{b^2} = 0$	2	2	
Parabolic Cylinder	*x*^2^ + 2*rz* = 0	3	1	
Elliptic Cylinder (imaginary)	$\frac{x^2}{a^2} +\frac{y^2}{b^2} = -1$	3	2	
Elliptic Cylinder (real)	$\frac{x^2}{a^2} +\frac{y^2}{b^2} = 1$	3	2	
Hyperbolic Cylinder	$\frac{x^2}{a^2} - \frac{y^2}{b^2}= -1$	3	2	
Elliptic Cone (imaginary)	$\frac{x^2}{a^2} +\frac{y^2}{b^2}+\frac{z^2}{c^2} = 0$	3	3	
Elliptic Cone (real)	$\frac{x^2}{a^2} +\frac{y^2}{b^2}-\frac{z^2}{c^2} = 0$	3	3	
Elliptic Paraboloid	$\frac{x^2}{a^2} +\frac{y^2}{b^2} =z$	4	2	–
Hyperbolic Paraboloid	$-\frac{x^2}{a^2} +\frac{y^2}{b^2} = z$	4	2	+
Ellipsoid (imaginary)	$\frac{x^2}{a^2} +\frac{y^2}{b^2}+\frac{z^2}{c^2} = -1$	4	3	+
Ellipsoid (real)	$\frac{x^2}{a^2} +\frac{y^2}{b^2}+\frac{z^2}{c^2} = 1$	4	3	–
Hyperboloid of one sheet	$\frac{x^2}{a^2} +\frac{y^2}{b^2}-\frac{z^2}{c^2} = 1$	4	3	+
Hyperboloid of two sheet	$\frac{x^2}{a^2} +\frac{y^2}{b^2}-\frac{z^2}{c^2} = -1$	4	3	–

For a point set $P = \{p_i\}^n_1$ drawn from a quadric surface, Eq. (2) can be extended into the shape *Ax* = 0, where *A* is the *n* × 10 matrix comprising the known point components, and *x* may be a column vector speaking to the obscure discriminant coefficients:

(4) $$\left[{\matrix { x_1^2  x_1y_1  x_1z_1  x_1  y_1^2  y_1z_1  y_1  z_1^2  z_1  1 \\ x_2^2  x_2y_2  x_2z_2  x_2  y_2^2  y_2z_2  y_2  z_2^2  z_2  1 \\ \vdots  \vdots  \vdots  \vdots  \vdots  \vdots  \vdots  \vdots  \vdots  \vdots \\ x_n^2  x_ny_n  x_nz_n  x_n  y_n^2  y_nz_n  y_n  z_n^2  z_n  1 }}\right] \left[ {\matrix {a \\ b \\ c \\ d \\ e\\ f \\ g \\ h \\ k \\ j }} \right] = 0$$

## Methodology

In this section, we explained the methodology of our proposed compression and decompression technique. The idea is to filter the point cloud to reduce the effect of noise when surface coefficients are computed to compress a point cloud. Thus, after filtering the noisy points, segmentation is performed and for each segment, quadric surface coefficients are computed along with boundary conditions. These coefficients and the boundary conditions are later used to decompress the point cloud. A more detail discourse is given in this section along with pictorial illustration of each step.

### Compression technique

**Figure 1:**
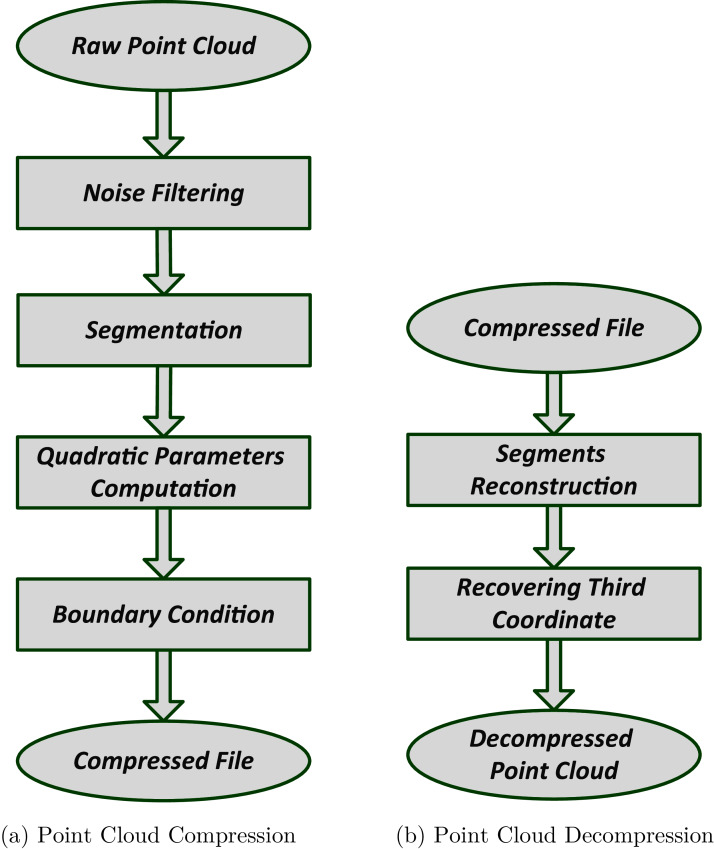
Point cloud compression and decompression block diagram.

The compressed version of the point cloud contains a set of surface’s coefficients and their respective boundary conditions. The block diagram to compute the surface coefficient and to find out the boundary conditions is given in the Fig. 1A.

### Filtering

Raw point cloud contains noise due to various sensor-specific and scene-specific limitations when acquiring the point cloud data (Ahmed et al., 2015). Therefore, suppressing the effect of noise is an essential step to avoid any incongruity. Voxel Grid (Munaro, Basso & Menegatti, 2012), Conditional Removal (Lim & Suter, 2007), and Statistical Outliers Removal (Rusu & Cousins, 2011) are commonly available filters to reduce the effect of noise from 3D point cloud datasets.

**Figure 2:**
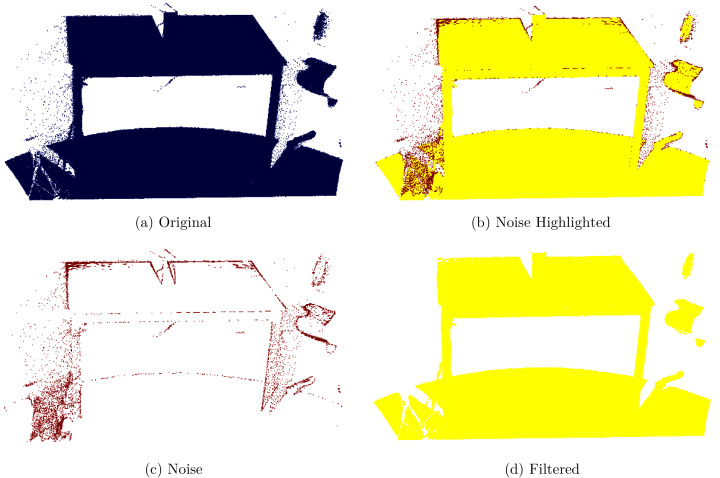
Original and filtered point cloud. (A) Original, (B) noise highlighted, (C) noise, (D) filtered.

Statistical outlier removal filter is used in this research because of its computational efficiency (Schall, Belyaev & Seidel, 2005). Figure 2A shows a noisy point cloud as captured from the sensor, Fig. 2B highlights the noise, Fig. 2C noise is given, and Fig. 2D a filtered point cloud is shown. This filter performs statistical analysis on each point by considering the point spread in the neighborhood and remove those points that could not meet the smoothness criterion. The mean distance from each point to its neighbors is computed under the assumption that resulted distribution is Gaussian with a mean and standard deviation. Once the distribution is computed, it is trivial to verify each point on an interval defined by global distance mean and standard deviation.

**Algorithm 2:** 3D point cloud decompression.

Require: Original Point Cloud {*C*} Just for Computing RMSE
Require: Compressed File {*CF*} of *C*
1: for each Plane *p* in *CF* do
2: Generate {*Points*} under the Concave-hull {*CH*}
3: for each *point* in *Points* do
4: Recover missing coordinate by applying Planer Equation *ax* + *by* + *cz* + *d* = 0
5: end for
6: Add this segment to Reconstructed Point Cloud {*RPC*}
7: end for
8: for each Quadric Surface *q* in *QL* do
9: Generate {*Points*} under the Concave-hull {*CH*}
10: for each *point* in *Points* do
11: Recover missing coordinate by applying Quadratic Eq. *ax*^2^ + *by*^2^ + *cz*^2^ + *dxy* + *exz* + *fyz* + *gx* + *hy* + *iz* + *j* = 0
12: end for
13: Add this segment to Reconstructed Point Cloud {*RPC*}
14: end for
15: Decompression Time per point {*DT*} = *Time*/Size of *C*
16: Average *RMSE* = {*RMSE* from *RPC* to *C* + *RMSE* from *C* to *RPC*}/2
17: Display Average *RMSE* & *DT*

### Segmentation

A filtered point cloud is then processed to divide the point cloud into several clusters within a degree of smoothness. The quadric representation of smoother surfaces is more stable therefore clustering smooth surfaces is performed before computing the quadric coefficients. Several methods are available to extract areas of a point cloud such as surface splatting (Zwicker et al., 2001), multi-level partitioning of unity implicit (Ohtake et al., 2003), and region growing segmentation (Rabbani, Van Den Heuvel & Vosselmann, 2006). Region growing approaches exploit the important fact that points which are close together have similar curvature values. Region growing segmentation is used in this work, it is based on a smoothness constraint defined on surface curvature of a small neighbourhood.

The surface curvature *γ* for a point *p* employing a small neighborhood is computed as the proportion of the biggest to the sum of the three eigenvalues of the co-variance matrix. Neighboring points are combined as a region in the event that they are comparative sufficient beneath a characterized smoothness imperative.

To begin with, all points are sorted by their curvature values. The region growing handle starts from the least curvature point which is utilized as a seed point because it dwells within the flattest region. For each seed point, the algorithm finds a bolster region around the seed.

1. Each neighbor point is tried for the angle between its normal and the normal of the current seed point. In case the angle is less than a limit at that point the current neighbor point is included to the current region. This avoids corners and edges from being included in the current region.
2. In case the surface curvature *γ* of the neighbor point is less than a limit, it is included to the set of seeds. This makes a difference to develop the region beyond the current neighborhood of the seed point. On the opposite, in the event that the surface curvature is over the limit, such points are not included in the set of seeds. It is taken to note that such points are ordinarily having a place to the corners or edges.
3. When all the neighbors of the current seed point are tried, the seed point is expelled from the set of seeds.

The above steps are repeated until all points have been tested and there is no more seed point remaining. The output of the algorithm is a set of regions. After region growing, points with large curvature values which are not the part of any region are considered as noise and discarded. Each region is processed further to find out the quadratic coefficients.

### Surface representation

The segments extracted from the segmentation phase are analyzed such that surface coefficients are computed for each segment. The abundance of non-planar surfaces in the natural environment leads us to the use of a generic representation. Quadric surface equations can represent a variety of non-planar segments captured using a 3D range sensor as given the Table 1. Section “Quadric Surface Representation” discusses the concept of computing quadric surface coefficients from a point cloud segment. Thus, a segment is either represented by a planar surface or a quadric surface based on the geometry of the segment. Quadric surface is represented by ten coefficients as given in Eq. (4) if a surface is non-planar. If the surface is pure planar, it can be represented by a polynomial of degree one in three variables. Hence, only four coefficients can represent such a segment. Therefore, a compression algorithm needs to use the representation which requires fewer coefficients where possible. The quadratic surface coefficients are also computed by fitting the equation to a quadric surface in the least square approximation fashion. Hence, each surface is first checked, if it can be represented by a plane and its plane fitting error is less than quadratic error, than only four coefficients can be used to represent it. To represent a surface in a planar form Eq. (4) can be simplified as

(5) $$dx + fy + kz + j = 0$$

where *d*, *f*, *k*, and *j* are plane coefficients. Once, the plane and quadratic coefficients of a segment are computed, each segment must pass the following tests to be admitted for the next phase.

1. Points in each plane must be close together within a certain radius to form a single cluster such that neighboring points must be within a predefined cluster tolerance distance to avoid any incorrect plane extraction. If more than one cluster exists, then each cluster is treated separately to fit a plane.
2. Each segment is evaluated using eigenvalue decomposition such that the smallest eigenvalue must be relatively small compared to the other two, to indicate that the points lie in a plane.

If the segment plane fitting error is greater than quadratic error, it means the segment does not pass the verification test explained in Algorithm 1 and the segment is considered as a non-planar surface. To cater to the non-planar surfaces, polynomial equations of degree two are preferred which can preserve surface geometry of curved and complex shapes. Quadratic equations are non-linear equations thus can capture the non-linearity of the surface.

**Algorithm 1:** 3D point cloud compression.

Require: Point Cloud: {*C*}
1: Apply Statistical Out-lier Removal Filter on *C*
2: Calculate Point Normals {*N*}
3: Apply Region Growing Segmentation and Generate List of Regions {*R*}
4: for each Region *r* in *R* do
5: Apply *RANSACQSFit* on *r* and Estimate Planer Coefficients *a*, *b*, *c*, *d* and Quadratic Coefficients *m*[0] to *m*[9]
6: Compute Planer Error {*PE*} and Quadratic Error {*QE*}, Concave-Hull {*CH*}, Eigenvalues *λ*_1_, *λ*_2_, *λ*_3_ of *r*
7: Discard minimum variance coordinate of *r*
8: Preserve Two Dimensional *CH* in Compressed File {*CF*}
9: if *PE* < *QE* then
10: Preserve Planner Coefficients *a*, *b*, *c*, *d* in *CF*
11: else
12: Preserve Quadratic Coefficients *m*[0] to *m*[9] in *CF*
13: end if
14: end for
15: Compression Percentage: {*CR*} = Size of (*C* − *CF*/*C*) * 100
16: Compression time per point {*CT*} = *Time*/Size of *C*
17: Display *CR* & *CT*

### Boundary conditions

A surface is represented by a quadric surface coefficients with initial boundary limits at infinity. However, to limit the surface in its actual form, boundary conditions are necessary. These conditions are, later, utilized in the reconstruction process of segments in the decompression phase.

It is worth mentioning here that shape, size, and area of the reconstructed objects are maintained when boundary conditions are known. Thus, an individual segment is an input to this module and it returns the boundary conditions in the form of three-dimensional points of lower and upper bounds.

To compute the boundary condition of a quadric surface, we analyze the geometry of the surface. It is important to note here that the surface segments from the region growing segmentation process, explained in Section “Segmentation”, are 2.5D surfaces thus can be projected onto a plane. Therefore, we compute the eigenvalue decomposition of a surface. The spatial coordinates within a local neighborhood are exploited to derive a 3D covariance matrix. The three eigenvalues *λ*_1_, *λ*_2_ and *λ*_3_ are non-negative and their magnitude indicate corresponding eigenvector (Dittrich, Weinmann & Hinz, 2017). From eigenvalues we can categorize a surface as follows:

1. **1D** Linear structure having points spread along one of the three axes, if $\lambda_1 \gg \lambda_2, \lambda_3$.
2. **2D** planar structure points spread along two of the three axes, if $\lambda_1, \lambda_2 \gg \lambda_3$.
3. **3D** points spread along all three axes, if *λ*_1_, *λ*_2_, and *λ*_3_ are approximately similar to each other.

In our case, we normally find surfaces in the last two categories. Our objective here is to project the points on a plane. For the 2D case, the points are already on a plane. For the 3D case, the axis with minimum eigenvalue is discarded and the other two axes are preserved. The eliminated axes can be retrieved through calculations discussed in the decompression Section “Decompression”.

Once the quadric surfaces are projected on a plane. The next step is to find the boundary of each surface. To compute the boundary of a 2D surface. We used a technique to find the *α*-concave hull of the surface given by Asaeedi, Didehvar & Mohades (2017). The *α*-Concave Hull is a generalization of the convex hull to compute the region occupied by a set of points. The 0-concave hull is equal to the convex hull of points of the surface and the 12-concave hull is a semi-convex hull. For $\alpha \gg 100$, the α-concave hulls construct sharp angles and the 180-concave hull is equal to the simple polygon with minimum area that contains all points of the surface. So the default is 180-concave where sharp angles can be maintained so the shape of the surface is retained as is. The *α*-concave hull of a set of points has the following attributes:

1. The *α*-concave hull is a simple polygon.
2. The *α*-concave hull includes all points.
3. All internal angles of the *α*-concave hull are less than 180+*α*.
4. The area of *α*-concave hull is minimal.

**Figure 3:**
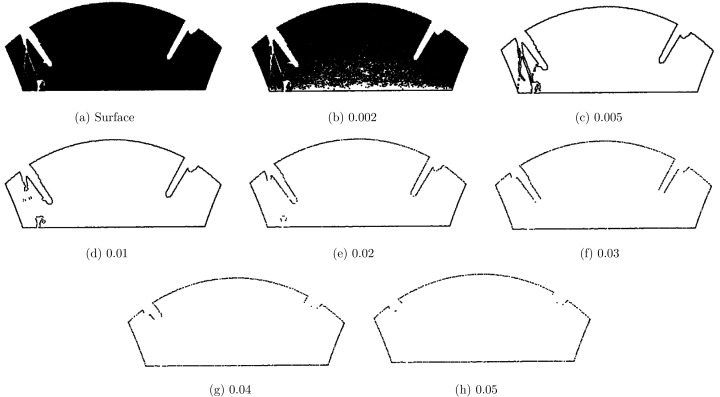
(A–H) Concave Hull at different α values.

The area of the *α*-concave hull is minimal thus it preserves the optimal boundary of the quadric surface. The variable *α* is a refinement parameter for the boundary of a surface. The change in *α* value and the impact on the boundary of a surface is illustrated in Fig. 3. As the value of the alpha *α* decreases, we see the boundary becomes smooth and abrupt changes in the boundary are reduced. However, if the value of *α* is very large, it can be noticed that the boundary becomes irregular and it tries to include unnecessary points into the boundary. Thus, *α* parameter plays a significant role to maintain the structure of the surface. The results of the boundary condition is a set of boundary points for each surface.

### Compressed output

The increase in the use of the 3D point clouds in a variety of fields, such as graphics, autonomous vehicles, head-mounted displays, requires a large amount of memory to store and bandwidth for transferring over the Internet. Applications demand efficient storage, processing, and transmission of 3D data.

The compression process is motivated by the work presented by Navarrete et al. (2016). Where the process is significantly similar, however, we found that using quadric representation instead of mesh representation significantly improves the compression as well as the computational performance of the algorithm.

The flow of the compression algorithm is given in the flow chart, illustrated in Fig. 1A. A raw point cloud is passed through a noise filter which in our case is a statistical outlier removal filter used to reduce outliers.

A surface-level representation of the refined point cloud is extracted using region growing segmentation. The quadric surface coefficients for each surface are computed. The boundary conditions for the surfaces are extracted using *α*-concave hull technique. The planar surfaces are segregated from all other surface types because planes require only four coefficients.

Finally, a compressed file of the 3D point cloud is generated that contains, surface coefficients, boundary conditions, and header information about the number of segments. The compressed file is significantly smaller in size as compared to the original point cloud. The information stored in the compressed file is then used to decompress the file in the decompression phase.

### Decompression

The decompression or reconstruction process of the proposed compression technique is shown in the flow chart, given in Fig. 1B.

The compressed file is composed of the properties of surfaces such as surface coefficients, boundary conditions, and other primitive information about the surfaces in the header.

The first step is the generation of points using the surface properties. To do so, the properties of each surface are processed to check the total number of points present in each surface at the time of compression such that an equal number of points can be generated as of the original surface. Some surfaces contain holes, for such surfaces, two or more boundary conditions are maintained. The point generation processes modified accordingly as it generates points under the upper/main concave hull and outside of the inner/hole concave hulls.

Once the projected shape of each surface is reconstructed, the next step involves the estimation of the third missing coordinate of each point, which is calculated by quadratic surface representation. If the surface type is planar, then only four planar coefficients are used to extract the third coordinate.

In the case of the quadratic equation, similarly, 2D points are generated under the *α*-concave hull and the third coordinate is estimated by the quadratic equation. The number of roots of a polynomial equation is equal to its degree. Hence, a quadratic equation has two roots. To use one of the two roots, both roots are verified using the planar equation of the projection of the quadric surface. The root that minimizes the fitting error is used for further processing.

**Figure 5:**
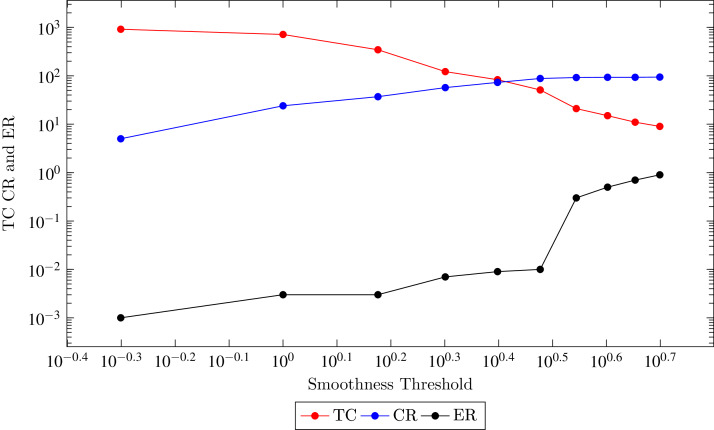
Total clusters (TC), compression ratio (CR) and error rate (ER).

The decompressed point cloud is available once the third coordinate is successfully computed. It is important to note here that the number of points after decompression is approximately similar to the original point cloud. However, the proposed technique is fully capable to reconstruct a denser or a coarser point cloud. Figure 5 shows the reconstruction of a point cloud at different densities percentage of the actual number of points in the original point cloud. This feature is very useful for some applications where a piece of structural level information is enough thus a coarse reconstruction is required. On the other hand, minute details of the captured object can be depicted using dense reconstruction.

## Experimental Results

The dataset utilized in this research is very comprehensive and categorical to compare with state-of-the-art compression techniques. The categories of the data are divided into structured and textured scene that are real and synthetic. The point clouds in this dataset are captured by Kinect 3D sensors, some are from TUM RGB-D benchmark and synthetics are generated from own rendering tool for simulation using java 3D. These datasets were published in Navarrete et al. (2016) and online available at Dataset complexity is classified into three classes such as High, Medium, and Low structured. High structured means architectural scene with mostly planar surfaces. Medium structured is a mixture of both architectural and real scenes whereas low structured is where the minimal planar information is present. We compared the proposed technique with a number of state-of-the-art algorithms for compression and decompression available in the literature. Before presenting the comparison, a brief description of the parameter optimization is useful.

## Parameter Optimization

### Statistical outlier filter threshold

Due to various sensor-specific and scene specific reasons the depth measurement of the sensor are erroneous. Therefore, it is an essential to pre-process the data in order to achieve optimal results. One of the pre-processing steps is noise filtering. We used the Statistical Outlier Filter to reduce the effect of sensor and scene specific noise. The Statistical Outlier filter computes statistical distribution of points with a mean and standard deviation. The distance of each point from its neighbors is filtered using a standard deviation threshold to qualify it or to discard it. Thus, one of the core parameters of the noise filtering method is standard deviation threshold.

### Smoothness threshold

**Figure 7:**
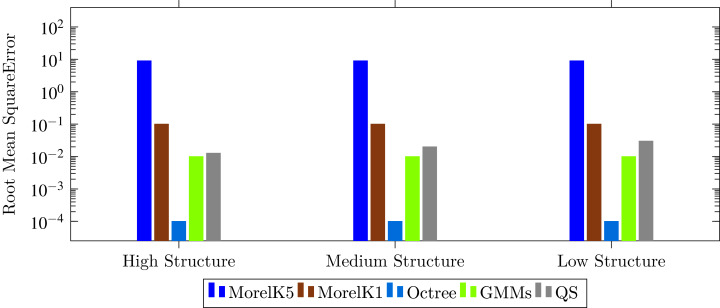
Comparison of proposed technique with state-of-the-art techniques with respect to root mean square error.

Region growing segmentation technique computes principle curvature of all the points and sort them in an increased order. The point with minimum curvature value is selected as a seed of first cluster. The smoothness of a seed with its neighbouring points is computed. The region grows by adding neighborhood points of seed if they meet the smoothness criterion. The segment formation process iterated until all the points are been the part of any segment. The smoothness threshold directly affects the reconstructed surface error and compression rate. The smoothness threshold is directly proportional to the compression rate. However, inversely proportional to error rate as shown in the Fig. 7. As the smoothness threshold decreased, number of segments increased and due to overhead cost of each segment less compression rate achieved but Quadratic Error (QE) decreased as well. From the empirical analysis on low and high structured datasets, surface smoothness threshold 3 degree is better to achieve both goals of high compression and less error rate. This value is also proposed by Point Cloud Library (*PCL*) as a standard.

### RANSAC QFit parameters

Random Sample Consensus Method for Quadratic Fitting is used to find the best quadratic coefficients of a surface. The first step in this process is to pick random points from surface and find quadratic coefficients *a* to *j* of Eq. (6) by applying least square method.

(6) $$ax^2+by^2+cz^2+dxy+exz+fyz+gx+hy+iz+j=0$$

This process is repeated and each iteration is executed with the purpose to find coefficients that having minimum QE which can be computed by Eq. (6). Experiences show that with 1000 iterations quadratic equation coefficients are computed.

### α Values

*α*-concave hull is to find the boundary conditions or the outline of a surface, and the *α* value strongly affects as discussed in “Boundary Conditions”. For *α* = 0 gives a convex hull and *α* = *π* is a maximum area polygon. Figure 3 illustrate surface outline for different values of *α*. Figure 3A is a complete surface and Figs. 3B to 3H are showing the affect of *α* variations on surface boundaries. At a very low *α* value 0.002, the concave hull is like original surface as shown in the Fig. 3B For a higher *α* value, gaps are visible on the boundary which highly affect the surface reconstruction. We conclude from experiments that *α* = 0.02 is useful value to detect boundary conditions on different structured datasets.

## Results

A dataset, consists of 101 point clouds of both real and synthetic scene, provided by Morell et al. (2014) is used in this research to compare the effectiveness of the proposed compression method. We performed 100 experiments for empirical analysis to fine tune the parameters such as RANSACQF, RANSAC segmentation, and *α*, as discussed in the Section “Parameter Optimization”. We write our code in C++ language by using Point Cloud Library (PCL).

**Figure 6:**
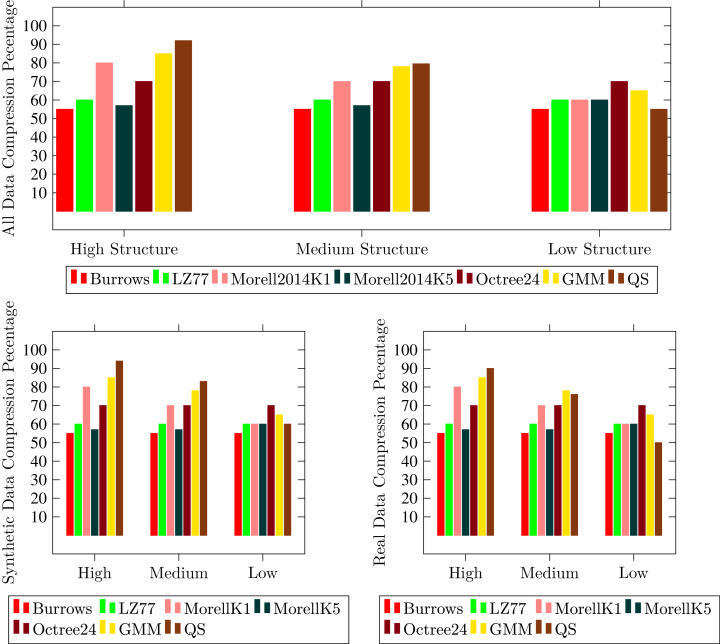
Comparison of proposed technique with state-of-the-art Lossy (Morell k1, Morell k5,Octree24 and GMM) and Lossless (Burrows and LZ77) techniques with respect to compression ratio.

**Figure 8:**
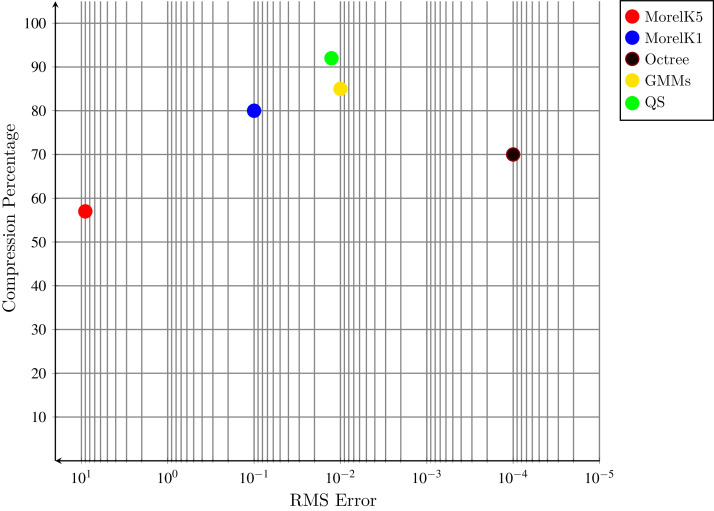
Compression percentage and RMSE graph.

**Figure 9:**
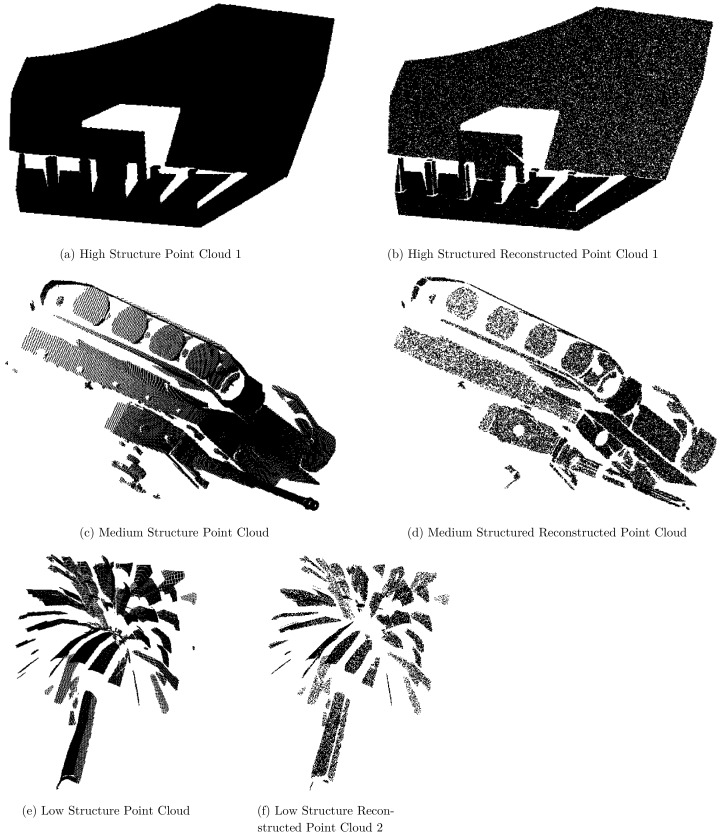
(A–F) Original and reconstructed high, medium and low structure point clouds.

The results are evaluated on the basis of three measures; Compression Ratio (CR), Root Mean Square Error (RMSE) and runtime to compress and decompress point cloud. We compared the proposed technique with state-of-the-art compression techniques of both lossless and lossy compression types. Two lossless compression techniques; LZ77 (Ziv & Lempel, 1977; Burrows & Wheeler, 1994) and four lossy techniques; Octree24 (Kammerl et al., 2012), Morell2014k1, Morell2014K5 (Navarrete et al., 2016), and GMMs (Navarrete, Viejo & Cazorla, 2018) are evaluated using the same measures to perform a fair comparison with the proposed technique. The comparison is classified according to the structure of point clouds and shown in the Figs. 6–9.

Lossless techniques LZ77 (Ziv & Lempel, 1977; Burrows & Wheeler, 1994) achieve reasonable compression rate (Up to 60%) with neither color nor structure error. These are performed better on real point clouds which consist of a large number of points as compared to synthetic point clouds that have few thousand points. Binary method to store point clouds occupy less storage as compared to American Standard Code for Information Interchange but still their memory requirement is a major penalty. Researchers continued research to achieve higher compression rate and compromised on error rate.

Lossless techniques LZ77 (Ziv & Lempel, 1977; Burrows & Wheeler, 1994) achieve reasonable compression rate (Up to 60%) with neither color nor structure error. The performance of these techniques is better on real point clouds. The techniques resulting in binary output to store a point cloud are slightly better than those store ASCII information. However, the compression is still requires further maturity. Therefore, a large number of researchers all around the globe are investigating to achieve higher compression rate with an acceptable on error rate.

Octree based compression have a linear relationship between compression rate and the number of planes in data. A scene with no planes has a lower compression rate as compare to a scene with a higher number of planes. This method could achieve up to 70% compression (Elseberg, Borrmann & Nüchter, 2013) as shown in the Fig. 8. One of the major drawbacks of this method is that once the point cloud is reduced it could not be able to regenerate the erased points. Geometric method given by Morell et al. (2014) with *k* = 1 and *k* = 5 is lossy compression method. Former is fast and latter is slower to achieve high compression. It uses environmental information mostly planes to get higher compression but its error rate is also high. In organized point clouds points are close to each other in 3D space and this method take the advantages of this feature. Overall compression in Morell et al. (2014) on desired dataset is 80% and synthetic point clouds having higher compression then real point clouds.

The latest advancement in point clouds compression is given by Navarrete, Viejo & Cazorla (2018). The Gaussian Mixture model is used to compress the point clouds. GMM achieved up to 85.25% compression rate with 0.01 decompression root-mean-square error per point. The computational expense of this method is mostly due to extraction of planes.

Our proposed compression technique enhance the plane extraction concept and introduce quadric surfaces detection and their representation with quadratic equations to compress and decompress point clouds. Figures 8 and 9 showing the results of Compression Ratio and Error Rate respectively. We have tested several data sets but it is very difficult to show all the properties of each point cloud. However, all properties of five point clouds of each class are presented in Table 2. The experiments show that in highly structured point clouds our proposed compression method achieved up to 91.17% compression ratio with less RMSE 0.01 per point within acceptable compression and decompression time 13.617 and 18.77 μs respectively.

**Table 2:** Performance measures of proposed technique of multiple classes of point clouds.

Category of dataset	Name of point cloud	Size (KB)	Number of points	Compressed file size (KB)	Compression ratio (%)	Compression time per point (*μ*s)	Decompression time per point (*μ*s)	RMSE per point	Compression total time(s)	Decompression total time(s)
High structure	hs_1	4,664	298,463	244.86	94.75	10.424	22.632	0.0142	3.111	6.754
	hs_2	2,971	190,073	182.13	93.87	13.532	16.335	0.0211	2.572	3.104
	hs_3	3,565	228,141	344.37	90.34	16.654	14.275	0.0070	3.799	3.256
	hs_4	4	210	0.49	87.56	18.134	15.980	0.0058	0.003	0.003
	hs_5	2,719	174,023	240.08	91.17	9.3450	24.632	0.0037	1.626	4.286
	Average				91.17	13.617 *μ*s	18.770 *μ*s	0.0103	2.219 s	3.476 s
Medium structure	ms_1	1,151	73,637	240.40	79.11	16.245	13.762	0.1352	1.196	1.013
	ms_2	2,376	152,042	321.03	86.49	19.853	21.643	0.1553	3.018	3.290
	ms_3	2,429	155,430	628.49	74.13	17.643	10.123	0.2532	2.742	1.573
	ms_4	4,801	307,200	587.44	87.76	22.654	14.277	0.0234	6.959	4.385
	ms_5	1,186	75,875	232.50	80.40	23.743	25.148	0.3424	1.801	1.908
	Average				81.58	20.027 *μ*s	16.990 *μ*s	0.1819	3.143 s	2.433 s
Low structure	ls_1	4,208	269,268	915.34	78.25	22.373	24.765	0.2102	6.024	6.668
	ls_2	1,263	80,813	412.53	67.34	16.288	22.562	1.6636	1.316	1.823
	ls_3	905	57,905	325.34	64.05	23.196	22.174	1.5436	1.343	1.283
	ls_4	1,367	87,455	354.12	74.10	24.875	23.162	0.0051	2.175	2.025
	ls_5	1,782	114,029	735.92	58.70	16.743	17.288	0.0124	1.909	1.971
	Average				68.49	20.695 *μ*s	21.990 *μ*s	0.6869	2.553 s	2.754 s

These results are better than the state-of-the-art techniques for most datasets, a comparison is shown in the Table 3. One of the main reasons of better results is the use of quadric surface representation. Most state-of-the-art techniques are focused on planes processing to compress point clouds. In our case, we use quadratic equations to represent quadric surfaces, which consists of multiple planes. Thus using lower parametric information to store a point cloud. Use of the quadric surface representation for point cloud compression is our main contribution to the field of computer vision.

**Table 3:** Porposed technique polynomials of degree two comparison with state of the art techniques.

Evaluation parameter	Burrows & Wheeler (1994)	Octree Schnabel & Klein (2006)	Octree Elseberg, Borrmann & Nüchter, 2013	Morell k5 Morell et al. (2014)	Morell k1 Morell et al. (2014)	Dalunay Triangles Navarrete et al. (2016)	GMMs Navarrete, Viejo & Cazorla, 2018	Polynomials of degree one Imdad et al. (2019)	Polynomials of degree two
Compression ratio (%)	30	43	50	59	81	85	85.25	89.15	91

On medium structured point clouds, compression rate is better than the state-of-the-art techniques for synthetic datasets while very close to GMM for real datasets. However, on low structured point clouds our results are lower in terms of compression while maintaining better error rate. This is due to the fact that low structured datasets are mostly composed of small non-planar segments. Thus increasing the number of parameters to be saved to maintain the structure of the reconstructed point cloud. It has been noticed that in real scenarios mostly point clouds are composed of structured contents. For example, outdoor scenes like streets, buildings walls, roofs, windows, etc. and indoor cases like tables, chairs, and other objects are mostly high structured, and in rare cases we have to deal with complex structured datasets.

Figures 9A and 9B illustrates the results of an original and reconstructed point cloud which is taken from Morell et al. (2014) dataset. The number of points in the reconstructed point cloud is equal to the original point cloud and their displacement is noticed in the form of RMSE per point. Figures 9C–9F depicting medium and low structured original and reconstructed point clouds. It can be visually inspected the reconstructed point cloud in decompression phase is significantly similar to the original point cloud. Despite the fact that there are many holes and small details in the original point cloud. The reconstructed point cloud maintained all the details.

**Figure 4:**
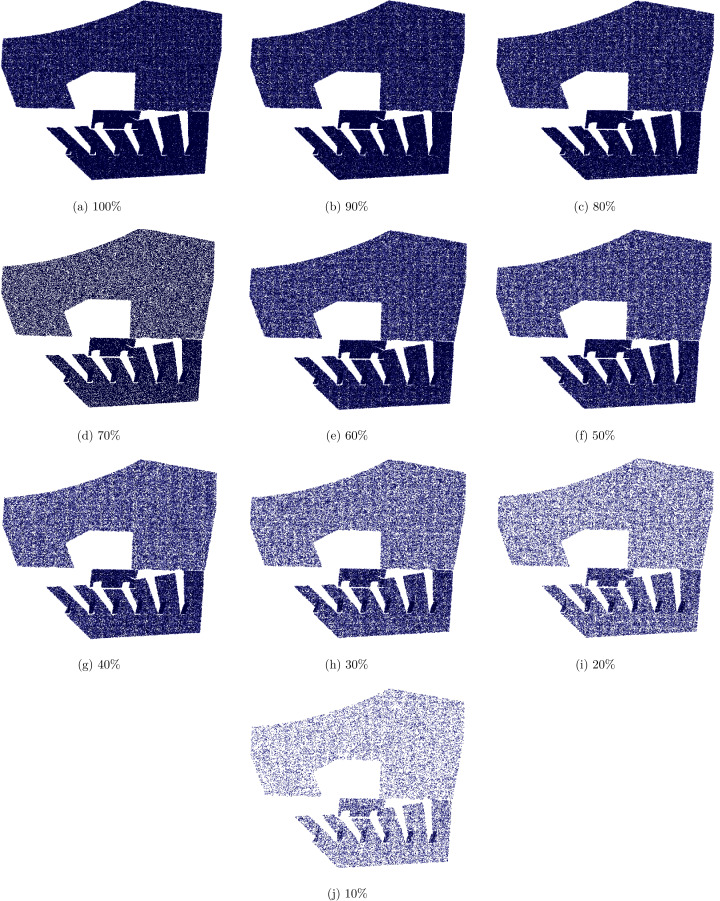
(A–J) A sample decompressed point cloud output on different density levels.

Another significant advantage of the proposed method is that the stored compressed file is in fact a vector representation of the scene. Thus, density of the point cloud is an adjustable feature. A sample point cloud was decompressed on different density levels from 100% to 10% in decreased order and shown in the Fig. 4 and computations are presented in the Table 4. A blue color is selected to show the points in the Fig. 4 and background is shown with white color. When the density level is high means 100%, the points inside and file size of decompressed file is equal to the original file. As the density level decreased, the overall structure of the point cloud is not destroyed but the distance between points is increased and the blue color darkness decreased. Initially, the increased distance between point is not visible on higher density levels because normally point clouds consists of hundred thousand points and their 90% or 80% are also a huge number of points. When the density the level is very low the distance between points increased which cause to decrease the quality of point cloud as shown in the Figs. 4I and 4J. Similarly, the effect of noise is reduced so the reconstructed point cloud is much smoother than the original point cloud, which may contain sensor and scene specific noise.

**Table 4:** A sample point cloud decompressed output on different density levels of Fig. 4.

Reference	Density (%age)	No. of points	Time in μs	RMSE
Figure 4A	100	254,467	23.421	0.0181
Figure 4B	90	229,200	23.462	0.0181
Figure 4C	80	203,743	23.414	0.0181
Figure 4D	70	178,310	23.386	0.0181
Figure 4E	60	152,840	23.405	0.0183
Figure 4F	50	127,352	22.986	0.0183
Figure 4G	40	101,875	23.426	0.0184
Figure 4H	30	76,393	23.475	0.0189
Figure 4I	20	50,927	23.513	0.0194
Figure 4J	10	25,567	23.445	0.0215

## Discussion

The proposed algorithm achieves a better compression rate compared to the state-of-the-art lossless algorithms as illustrated in the experiment section. The technique is based on surface extraction thus high structured scene has a more comprehensive representation. Similarly, the synthetic dataset is also well represented using surfaces because of no noise and well-defined edges and corners, thus the compression rate is higher compared to the other techniques. On medium structured objects, our results are comparable to the state-of-the-art. However, our technique struggles on the low structured scene because of two main reasons. (1) the low structured scene contains a significant amount of noise as depth sensors are not accurate on higher curvature areas such as corners and edges, and (2) fitting a quadric surface on a high curvature area is challenging thus algorithm tries to divide it into a large number of smaller smoother surfaces thus more computation is required. It has been noticed that most scenes in the urban settings are composed of highly structured content thus the algorithm can be used for comparison point clouds and transmit over mobile devices.

## Conclusion

In this research, we propose a novel lossy 3D point cloud compression and decompression algorithm based on geometric information of points, *i.e*., point normal and curvature values. We compress data by applying the Random Sample Consensus method for Quadratic Fitting to represent quadric surfaces with their respective quadratic coefficients and *α*-Concave hulls. We use a publicly available dataset to compare our results with state-of-the-art lossy and lossless compression algorithms to optimize memory and bandwidth requirements to store and transfer point clouds on a network within an acceptable computation time.

Experiment results show that the proposed method achieves a higher compression ratio and less *RMSE* compared to state-of-the-art lossy and lossless compression algorithms. The current method supports only point cloud data, thus colour information is not considered to further optimize the compression rate. In the future, we will enhance the capability of the proposed algorithm by exploiting the colour and texture information in the scene.

## Additional Information and Declarations

### Data Availability

The following information was supplied regarding data availability: The source Code is available at Zenodo: Muhammad Asif. (2021). Code and Data Set for PeerJ manuscript, “3D Point Cloud lossy compression using Quadric Surfaces” (57825) [Data set]. Zenodo. DOI 10.5281/zenodo.4740849. We used the Dataset in binary format available at 3DCOMET: http://www.rovit.ua.es/dataset/3dcomet/downloads.html.
